# Parenteral Nutrition, Sepsis, Acute Heart Failure and Hepatotoxic Drugs Are Related to Liver Test Disturbances in Critically Ill Patients

**DOI:** 10.3390/nu15112612

**Published:** 2023-06-02

**Authors:** Zenzi Rosseel, Pieter-Jan Cortoos, Joop Jonckheer, Wilfried Cools, Mathieu Vinken, Hendrik Reynaert, Elisabeth De Waele

**Affiliations:** 1 Department of Pharmacy, Universitair Ziekenhuis Brussel (UZ Brussel), Laarbeeklaan 101, 1090 Brussels, Belgium; zenzi.rosseel@uzbrussel.be (Z.R.);; 2 Department of Clinical Nutrition, Universitair Ziekenhuis Brussel (UZ Brussel), Laarbeeklaan 101, 1090 Brussels, Belgium; 3 Faculty of Medicine and Pharmacy, Vrije Universiteit Brussel (VUB), Laarbeeklaan 103, 1090 Brussel, Belgium; 4 Department of Intensive Care, Universitair Ziekenhuis Brussel (UZ Brussel), Laarbeeklaan 101, 1090 Brussels, Belgium; 5 Department of Support for Quantitative and Qualitative Research (SQUARE), Vrije Universiteit Brussel (VUB), Laarbeeklaan 103, 1090 Brussels, Belgium; 6 Department of Pharmaceutical and Pharmacological Sciences, Vrije Universiteit Brussel (VUB), Laarbeeklaan 103, 1090 Brussel, Belgium; 7 Department of Gastro-Enterology, Universitair Ziekenhuis Brussel (UZ Brussel), Laarbeeklaan 101, 1090 Brussels, Belgium

**Keywords:** hepatotoxic drugs, parenteral nutrition-associated liver disease, sepsis, acute heart failure, intensive care unit, liver disturbances

## Abstract

Background: Parenteral nutrition (PN) is often associated with liver dysfunction in the ICU, although other factors such as sepsis, acute heart failure (AHF), and hepatotoxic drugs can be equally present. The relative impact of PN on liver dysfunction in critically ill patients is largely unknown. Methods: We recorded the presence of pre-existing liver disturbances, AHF, sepsis, daily PN volume, and commonly used hepatotoxic drugs in adult ICU patients, together with daily aspartate aminotransferase (AST), alanine aminotransferase (ALT), gamma-glutamyltransferase (GGT), alkalic phosphatase (AP), total bilirubin (TB), and INR values in patients with three or more PN treatment days. A linear mixed-effects model was used to assess the relative contribution of each liver parameter. Nutritional adequacy was defined as intake/needs. Results: We included 224 ICU patients with PN treatment lasting more than 3 days between 1 January 2017 and 31 December 2019. For AST, pre-existing liver disturbances (+180% ± 11%) and the presence of AHF (+75% ± 14%) were the main predictors of deterioration, whereas PN volume caused only a limited increase of 14% ± 1%/L. Similar results were observed for ALT. GGT, INR, and TB are mainly influenced by the presence of sepsis/septic shock and pre-existing liver disturbances, with no impact of PN or hepatotoxic drugs. Carbohydrate intake exceeded recommendations, and protein and lipid intake were insufficient in this study cohort. Conclusions: Liver test disturbances in ICU patients on PN are multifactorial, with sepsis and AHF having the highest influence, with only limited impact from PN and hepatotoxic drugs. Feeding adequacy can be improved.

## 1. Introduction

According to the European Society of Parenteral and Enteral Nutrition (ESPEN) guidelines for nutrition in critical care, enteral nutrition is the first choice to cover nutritional requirements [1]. No difference in mortality has been found between the use of enteral versus parenteral nutrition (PN) at an intensive care unit [2]. Total parenteral nutrition (TPN) is preferentially indicated in patients with a dysfunctional gastrointestinal system, whereas supplementary parenteral nutrition (SPN) can be used in patients with a functional gastrointestinal system when nutritional requirements cannot be met with EN alone, e.g., in cases of prolonged ileus [3,4]. Correct dosing of PN is paramount, reduces mortality [5], and should be guided by the use of indirect calorimetry (IC). IC provides information about the resting energy expenditure (REE), which is used to guide nutritional prescription [6]. However, PN has been associated with liver dysfunction, with lipid emulsions in particular being considered culprits in the pathogenesis of PN-associated liver disease (PNALD) [7,8]. PNALD is characterized by cholestasis and necrosis due to liver inflammation, resulting in increased liver parameters, including alanine aminotransferase (ALT), aspartate aminotransferase (AST), alkaline phosphatase (AP), gamma-glutamyltransferase (GGT), total bilirubin (TB), and international normalized ratio (INR) [9]. Cholestasis causes an impairment in bile formation, resulting in an increase in AP and GGT [10], whereas an increase in the combination of AST, ALT, INR, and cholestatic enzymes (AP, GGT, or TB) is defined as a mixed pattern [7]. Although PN is not the sole contributing factor to liver disturbances in critically ill patients, sepsis can have negative implications for the liver due to a decrease in hemodynamic circulation [11,12]. Acute heart failure (AHF) can also trigger liver disturbances through hypoperfusion and congestion, inducing hepatocellular injury with an increase in AST and ALT [13]. Furthermore, numerous drugs are used to stabilize and improve patients’ health in intensive care units (ICUs), but these may negatively affect liver function through direct toxicity, toxicity of metabolites, or drug exposure time, leading to a drug-induced liver injury (DILI) [14,15]. Considering the multiplicity of factors contributing to liver disturbances, the aim of this study was to identify the individual impacts of PN, sepsis, AHF, and hepatotoxic drugs on liver parameters in ICU patients. These results can provide key messages to guide the treatment of patients with liver disturbances during PN therapy.

## 2. Materials and Methods

### 2.1. Study Design

A retrospective single-center cohort study on adult patients at the intensive care department of UZ Brussel, a tertiary care center in Brussels (Belgium), was set up. This study was approved by the Ethics Committee of the UZ Brussel (reference BUN 1432021000548).

### 2.2. Selection Criteria

A search was performed in the hospital electronic patient file system PrimUZ© (Version 2021.1.0, Brussels, Belgium) to compile two lists of patients admitted between 1 January 2017 and 31 December 2019. More recent data were avoided due to potential COVID-19 pandemic bias. The first list consisted of adult patients admitted for at least 3 days at ICU, while the second list included patients with at least 3 consecutive days of PN (3 components: lipids, carbohydrates, and amino acids). Both lists were combined to select patients eligible for this study. Exclusion criteria were <18 years, liver transplant, viral hepatitis, excessive alcohol consumption, short bowel syndrome, liver cancer, and cancer with liver metastases. Alcoholism was defined as >2 units/day (1 unit defined as 8 g of pure alcohol) or when the medical file included alcoholic steatohepatitis or alcoholic fatty liver disease. Selected patients were further divided into 2 groups, one with and one without liver test disturbances, defined as 2 out of 3 liver parameters (AST, ALT, or AP) being elevated at ≥1.5× upper limit of normal (ULN) at day 1 of PN. The separation of groups was done on the first day to see if the liver tests were elevated before the patient received PN.

### 2.3. Collected Data

For each patient, demographic data such as age, sex, height, and weight were recorded on admission, as well as length of ICU stay (LOS) and acute physiology and chronic health evaluation score (APACHE II). C-reactive protein (CRP) and liver parameters AST, ALT, AP, GGT, TB, and INR were recorded daily for a maximum of 10 days. Next to liver parameters, nutritional therapy was recorded daily. Patients’ nutritional requirements were calculated using ESPEN ICU guidelines (calories: 20–25 kcal/kg/day; proteins: 1.3 g/kg/day; carbohydrates: 2 g/kg/day; lipids: 1.5 g/kg/day) based on current weight (18.5 < BMI < 25) or adjusted body weight (ABW) (18.5 > BMI > 25) with ABW = ((actual body weight—IBW) × 0.33) + IBW; and IBW (ideal body weight) = 22 × (height in m)^2^ [16].

### 2.4. Co-Morbidities

As AHF and sepsis/septic shock can all have a negative impact on the liver, their occurrence was recorded during ICU admission. Diagnosis of sepsis was based on the presence of at least two of the following criteria: hyperthermia > 38.3 °C/hypothermia < 36 °C, tachycardia > 90 beats per minute, leukocytosis > 12.000/mm^2^, or tachypnea > 20 breaths per minute induced by bacterial infection. Septic shock was defined as the existence of sepsis in combination with vasopressor (dobutamine/norepinephrine/phenylephrine/epinephrine) therapy and lactate > 2 mmol/L [17,18]. AHF was defined as the presence of following diagnoses and/or drugs in the patient’s file: acute pulmonary oedema, AHF, cardiogenic shock, levosimendan, dobutamine, or Milrinone [19,20].

### 2.5. Hepatotoxic Drugs

To enable the evaluation of liver test abnormalities due to hepatotoxic drugs, a top 5 list of commonly used hepatotoxic drugs was defined based on the article by Björnnson et al. [14] and drug consumption at our ICU between 1 January 2019 and 31 December 2019. Amoxicillin/clavulanic acid, flucloxacillin, valproic acid (VPA), and sulfamethoxazole/trimethoprim (SMX/TMP) were selected because of their categorization in category A according to Björnnson et al., with >100—DILI cases reported within 92% of the cases as positive re-challenges. Paracetamol (PCM) was selected because of its known hepatotoxicity, which is responsible for approximately 50% of total DILI cases [21,22]. The final hepatotoxic drug list includes PCM, amoxicillin/clavulanic acid, flucloxacillin, VPA, and SMX/TMP. Each patient was screened for the administration of one or more of these drugs before or during ICU stay. Therapy duration, daily dose, and cumulative dose were recorded.

### 2.6. Statistical Analysis

Baseline characteristics were reported as proportions (%), mean ± standard deviation, or median [interquartile range] where applicable. To explain the observed liver parameter values, we used linear mixed effect models with random intercepts to accommodate the correlations implied by the repeated measurements. For each liver parameter (AST, ALT, GGT, AP, INR, and TB), we applied a natural log transformation to normalize residuals, and a best-fitting model was built based on the Akaike information criteria (AIC) using maximum likelihood (ML), and the final model was fitted with restricted maximum likelihood (REML), stepwise introducing various possible covariates. Age, sex, and BMI were also considered in the model. Other variables of interest were volume of PN administered daily, liver test disturbances at start of PN therapy, presence of AHF or/and sepsis/septic shock, and hepatotoxic drugs (cumulative dose of paracetamol, amoxicillin/clavulanic acid, flucloxacillin, valproic acid, sulfamethoxazol/trimethoprim, and treatment duration of paracetamol and amoxicillin/clavulanic acid). The number of days was the main covariate as it addressed changes over time, and it was always included in the model even though it may not necessarily lower the AIC. For each model obtained, a back transformation was performed afterwards to allow interpretation usable in clinical practice. Statistical analyses were performed using Statistical Package for the Social Sciences version 28.0 (IBM SPSS Statistics for Windows) and R version 4.1.3 package for the mixed model lme4, version 1.1.29 (R foundation for Statistical Computing).

## 3. Results

### 3.1. Study Population

**Figure 1:**
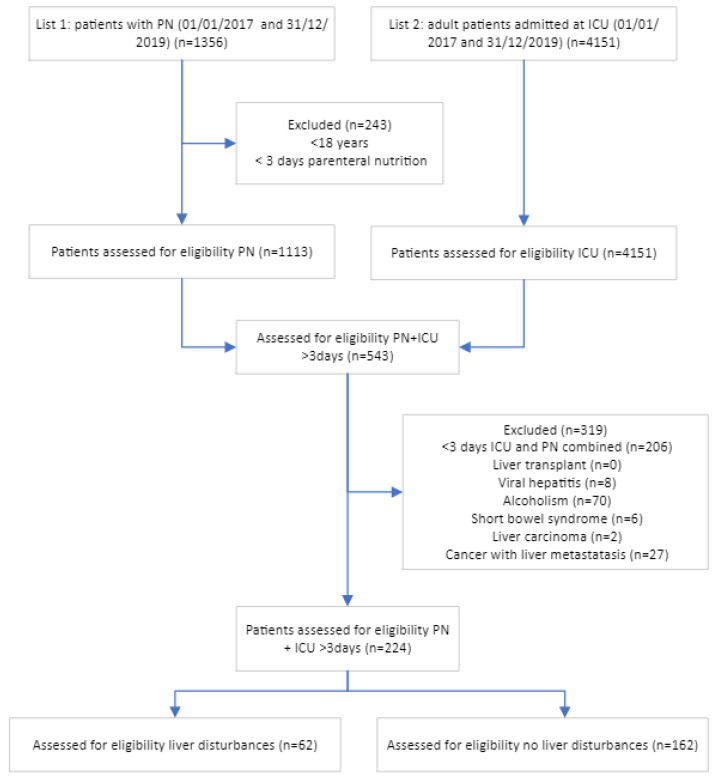
Enrollment and outcomes.

By combining the lists of ICU and PN patients, we obtained an intermediate list of 543 adult ICU patients receiving PN ≥ 3 days between 1 January 2017 and 31 December 2019 (Figure 1). After excluding patients not receiving PN at ICU for more than 3 days and patients with liver transplant, viral hepatitis, excessive alcohol consumption, short bowel syndrome, liver cancer, and cancer with liver metastasis, a final list of 224 patients remained, of whom 62 had liver test disturbances at the start of PN therapy and 162 did not.

The overall study population consisted mainly of overweight or obese (54.9% with BMI > 25) patients with a mean age of 63 ± 17 years and an ICU LOS of 19 [10–31] days. Patients received 8 [5–14] days of PN, and in 12%, indirect calorimetry was performed with a median REE of 1834 [1492–2139] kcal, corresponding to 25 [20,21,22,23,24,25,26,27,28,29] kcal/kg/day (mean weight patients with IC 73 kg).

Regarding patients with liver test disturbances at the start of PN therapy, significantly more patients suffered from sepsis (35.5% vs. 21.6, *p* = 0.033) and AHF (29% vs. 11.7%, *p* = 0.002), while the mean APACHE II score was higher (24 vs. 21, *p* = 0.048). When focusing on PN intake, the group with liver test abnormalities received more PN on a daily basis (1572 vs. 1450 mL, *p* ≤ 0.001), correlating with higher calorie (1767 vs. 1603 kcal, *p* < 0.001), amino acid (71 vs. 64 g, *p* < 0.001), carbohydrate (217 vs. 197 g, *p* < 0.001), and lipid (62 vs. 58 g, *p* < 0.001) intake. Hepatotoxic drug use was comparable in both groups (Table 1). The maximum daily dose administered of PCT was 4 g, 7 g for amoxicillin/clavulanic acid, 12 g of flucloxacillin, 3 g of valproic acid, and 8 g of SMT/TMP.

### 3.2. Liver Test Abnormalities Associated with PN, Drugs, AHF, and Sepsis

**Figure 2:**
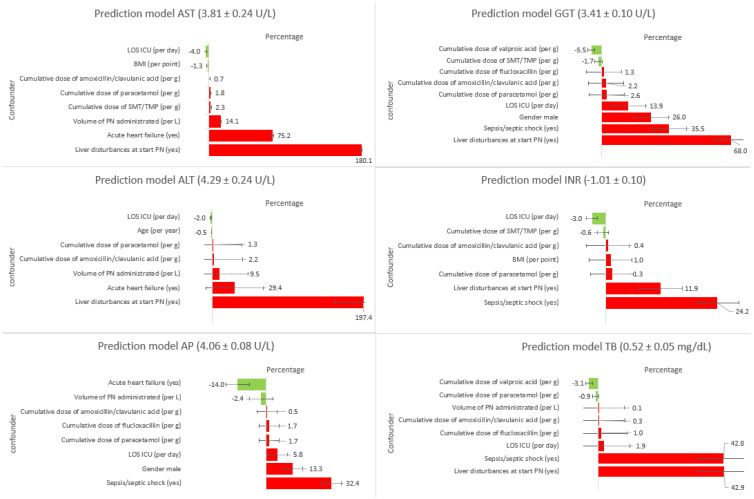
Prediction models illustrating the influence of confounders on all liver parameters.

Figure 2 shows the multivariable association between liver parameters and possible confounders. The largest impact on AST was seen with prior liver test disturbances at the start of PN therapy (+180.1% ± 11.2%), followed by the presence of AHF (+75.2% ± 13.7%). PN volume was also at the forefront and caused an additional increase of 14.1%/L ± 0.9% PN. PCM (+1.8%/g ± 4.1%), amoxicillin/clavulanic acid (+0.7%/g ± 0.5%), and SMX/TMP (+2.3%/g 1.0%) were important in predicting AST values, causing small increases compared with previous confounders. The presence of liver disturbances at the start of PN therapy caused an increase in ALT values (+197.4% ± 11.2%), followed by the presence of AHF (+29.4% ± 13.4%). Again, in this model, volume of PN was a contributing factor in predicting ALT (+9.5%/L ± 5.0% PN). Of the two morbidities recorded, only the presence of sepsis/septic shock had a negative impact on AP (+32.4% ± 8.2%). The presence of AHF had a protective effect on AP, causing a decrease of 14% ± 9.7%. Male sex was the second-most determining factor in predicting AP, with a 13.3% ± 7.4% higher AP compared with women. PCM, which normally affects AST and ALT, also affected AP with an additional increase of 1.7%/g ± 0.2% PCM.

Patients who were phenotypic for pre-existing elevated liver enzymes at the start of PN were observed to have the most detrimental increase (+68% ± 13%), followed by the presence of sepsis/septic shock (+35.5% ± 13.8%) in the model for GGT. The third important factor in predicting GGT was sex; male patents had a 26% ± 12% higher GGT value compared with women. Surprisingly, treatment with VPA (−5.5%/g ± 4.2%) and SMX/TMP (−1.7%/g ± 0.9%) caused a decrease in GGT values. The final model for INR showed evidence for several confounders, with sepsis having the biggest impact (+24.2% ± 4.9%), followed by liver test disturbances at the start of PN therapy (+11.9% ± 4.8%). Other confounders were significant in predicting INR values but had a minor impact (<3%).

For TB, the presence of liver disturbances at the start of PN therapy (+42.9% ± 6.3%), followed by the presence of sepsis/septic shock (+42.8% ± 6.6%), had the most detrimental effects. No other factors caused a big increase (>10%). On the other hand, treatment with VPA (−3.1%/g ± 2.0%) and PCM (−0.9%/g ± 0.2%) caused a decrease in TB values. Table A1 (Appendix A) presents the multivariable associations between all liver parameters and their possible confounders, whereas Table A2 (Appendix B) presents the multivariable associations between AST and possible confounders based on their biologically plausible association.

### 3.3. Feeding Adequacy

**Figure 3:**
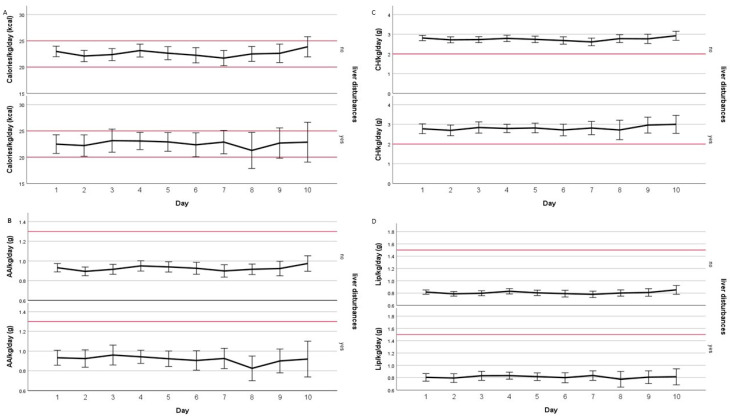
Evaluation of feeding adequacy according to ESPEN guidelines. Daily intake of (**A**): calories, (**B**): amino acids (AA), (**C**): carbohydrates (CH), and (**D**): lipids (Lip) received per kilogram of actual or adjusted body weight for a maximum of 10 days in both groups with and without liver dysfunction at start of PN therapy. Calories expressed as kcal/kg/day and amino acids, carbohydrates, and lipids as g/kg/day. Red line indicates recommendation of ESPEN: calories 20–25 kcal/kg/day, amino acids 1.3 g/kg/day, carbohydrates 2 g/kg/day, and lipids 1.5 g/kg/day. Lines represent the mean, and the whiskers represent the 95% confidence interval (CI).

All patients were treated with three chamber PN bags consisting of proteins, carbohydrates, and lipids. Enteral and parenteral intake was recorded daily, with a maximum of 10 days represented in Figure 3. Calorie intake was situated between 20 and 25 kcal/kg/day, which is in line with ESPEN guidelines. Lipid (0.8 vs. 1.5 g/kg/day) and protein intake (0.9 vs. 1.3 g/kg/day) were below recommendations, and carbohydrate load (3 vs. 2 g/kg/day) exceeded the guidelines during the whole follow-up (maximum 10 days). Caloric intake was sufficient because of the high carbohydrate intake (Figure 3). PN bag composition can be found in the supplementary file. Two hundred and seventeen patients (96.9%) received parenteral nutrition containing OO/SO 80/20%; only seven patients (3.1%) received a combination of SO/MCT/OO/FO (SMOF/schema A/schema C) with a ω6/ω3 ratio of 2.5:1. Regarding the infusion of lipids, a maximum of 80 g/lipids/day was parenterally administered during continuous infusion, resulting in a mean of 43.30 mg/kg/h. Patients receiving tube feeding also received continuous infusion at a maximum of 140 g/lipids/day, resulting in a mean infusion rate of 75.76 mg/kg/h. In any case, lipids were not administered at an infusion rate exceeding 110 mg/kg/h.

## 4. Discussion

In this retrospective study, we were able to demonstrate that liver test abnormalities were multifactorial and that the presence of pre-existing liver disturbances at the start of PN therapy had the highest impact on subsequent liver parameters, with the volume of PN playing only a minor role and the use of hepatotoxic drugs having a negligible impact.

In our study, the presence of sepsis appeared to be the most important factor contributing to liver test disturbances, to a much greater extent than PN use. A Spanish prospective study on 725 ICU patients using similar intravenous lipid emulsions (ILE) as our hospital already concluded that the presence of sepsis and treatment with PN were the main contributors to liver test disturbances, mainly cholestasis as well as mixed-pattern liver injury [7]. Another trial with 80 non-ICU patients and normal liver tests on admission showed, through multivariate association, that elevation of AP and ALT were associated with the presence of sepsis, and elevation of GGT was influenced by the amount of soy bean oil (SO) [23]. Lipid composition was partly comparable to ours, but their PN treatment duration was twice as long, while we observed no influence of the presence of sepsis on ALT. A large non-ICU trial included septic patients not treated with PN and concluded that sepsis-associated liver injury occurred in 35% of the patients with cholestasis [12]. Our results were in line with this trial: sepsis had a detrimental effect on GGT, TB, and AP. This illustrates the major impact of sepsis on liver function, regardless of pre-existing PN use (taking into account that PN was used short-term (8 [5–14] days)).

Sepsis and PN are not the sole contributing factors to liver test disturbances. A review from 2019 concluded that 20 to 30% of all patients show liver dysfunction due to acute heart failure (AHF). Patients often show few symptoms, but in laboratory tests, elevations up to 20× the ULN of aminotransferases can be observed [24]. Other studies [25,26,27] observed a predominant elevation in cholestatic enzymes or even a mixed pattern. In our study, AHF had a big impact on predicting aminotransferases, but no effects were observed for cholestatic enzymes. The results were in line with those of Xanthopoulos et al. AHF was the second-most important contributing factor in our retrospective analyses.

All patients in our study received at least 3 consecutive days of PN, with an average treatment duration of 8 days in the ICU, which can be considered short-term PN therapy. In our population, we observed only moderate aminotransferase changes (9–15% increase), theoretically indicating the presence of PNALD. Several non-ICU studies conducted in the 1990s demonstrated liver test abnormalities in hospitalized patients on long-term PN [28,29,30,31]. However, even after one week, enzyme abnormalities could be observed, both in cholestatic enzymes and aminotransferases [9,31]. Nevertheless, in a clinical setting, it is not always possible to distinguish the PN effect from other factors that are partially responsible for interfering with liver function testing, such as sepsis and AHF. The correlation between PN and disturbed liver function tests can be explained by the fact that, at the beginning of the 21st century, it became known that the fatty acid composition, especially SO, of ILE is a predictor for PNALD [32,33,34,35]. However, with the advent of olive oil (OO) and fish oil (FO) ILE, liver enzymes stayed within normal ranges, and these emulsion types were found to be safe and less associated with liver dysfunction [36,37], thus changing the paradigm. Patients in our study were predominantly fed with OO-containing PN (80/20% OO/SO), so cholestasis is expected to a lesser extent.

Lipids are necessary in both septic and AHF patients because fatty acids (FA) play a role in sustaining cell membranes and gene expression and have anti-inflammatory properties [38,39,40]. A sufficient lipid intake is clearly necessary, and excluding lipids from the diet will cause more harm to the patient and his recovery [38,39,41,42,43,44,45]. In our study population, lipid intake was below ESPEN recommendations (0.8 g/kg/day vs. 1.5 g/kg/day) [1]. A trial with 60 septic ICU patients investigated the effects of standard of care (SOC) (SOC was in accordance with surviving sepsis campaign guidelines) plus parenteral ω-3 polyunsaturated fatty acids (PUFA) compared with SOC alone. A significant reduction in organ dysfunction and CRP was observed in the ω-3 PUFA group and was associated with a reduction in mortality in patients with less severe sepsis [42]. Similar to AHF patients, administration of ω-3 PUFA was associated with fewer cardiovascular events and a lower mortality risk [44,45,46]. Different studies investigated the effects of OO and SO and concluded that due to the anti-inflammatory effect of OO (ω-9 monounsaturated fatty acid (MUFA)), a lower CRP was observed. This could be of benefit to septic patients at risk of developing septic shock as it could potentially reduce the overactivation of the inflammatory response, which will be the cause of shock and eventually multiple organ failure [47]. On the other hand, SO contains ω-6 PUFA and phytosterols (PY), which are known to be pro-inflammatory and could therefore be less recommended [39,43,46,48]. PY, the equivalent of cholesterol in plant-based oils such as SO, has been found to be detrimental for the liver, causing raised serum bile acid levels and decreased bile acid production, leading to cholestasis by antagonizing the farnesoid X receptor [49,50]. SO was, to a lesser extent, present in our PN regimens but cannot be fully excluded in ILE because it contains essential FA (linoleic acid and α–linoleic acid). Studies have assessed different types of ILEs, but there is a lack of data that has compared all available ILEs to draw conclusions regarding mortality. The ideal ratio of ω-3/ω-6 ratio in the ICU is not yet determined. The amount of SO needs to be decreased, but it is not excluded that the amounts of ω-3 and ω-9 need to be increased due to their lower inflammatory properties [51].

If ILEs are reduced or omitted, larger amounts of carbohydrates are needed and will lead to an increase in circulating insulin, contributing to increased lipogenesis and FFA production, which in turn will generate pro-inflammatory signals and can give rise to non-alcoholic fatty liver disease (NAFLD) with increased transaminase levels, typical for necrosis [52]. In another large prospective trial, a higher carbohydrate intake (>5 g/kg/day) was associated with the presence of cholestasis [53]. In our study, patients were administered on average 3 g/kg/day of carbohydrates (enteral and parenteral), far below the 5 g/kg/day from the study of Lakananurak et al. but still exceeding the ESPEN recommendation of 2 g/kg/day. This imbalance can be explained by an exaggerated carbohydrate load in the three chamber bags used in our setting, but we are unable to assess its impact on our patients since this study did not investigate dose relationships of the individual PN components.

Regarding the selected hepatotoxic drugs, paracetamol, amoxicillin/clavulanic acid, valproate, flucloxacillin, and sulfamethoxazole/trimethoprim, not all drugs contributed to each model for predicting liver parameters. These drugs had a minor impact (<3%) in all prediction models. Possible negative influences of these drugs mostly disappear into the background when sepsis, AHF, and PN are present. In contradiction to the other selected drugs, paracetamol has an especially detrimental effect on overdosing (6–10 g/day for several days) [54]. None of the study patients received daily doses exceeding 4 g.

This study had several limitations. Patients could have had sepsis/AHF before the start of PN treatment or developed AHF or sepsis/septic shock during PN treatment, which was not accounted for. Late-onset (>14 days–6 months) hepatotoxicity could not be detected because follow-up was provided up to a maximum of 10 days. Patients with non-alcoholic steatosis hepatitis (NASH) or gallstone pancreatitis were not excluded, nor were patients with intra-abdominal infections. AST, GGT, and TB are not liver-specific and could therefore be influenced, e.g., by hemolysis and muscle fraction. Regarding the top five selected hepatotoxic drugs, only drug use in 2019 was assessed to build the list, while study data was collected from 2017 to 2019. Moreover, the impact of other drugs was not assessed. No glycemic parameters were recorded, so nothing can be concluded regarding insulin resistance and its impact on liver parameters. Non-food calories such as infusions of glucose and propofol were not included in the analysis, leading to a possible underestimation of carbohydrate, lipid, and thereby total energy intake. The trophic effect of trickle feeding was not taken into account. Finally, PN regimens in our study were higher in carbohydrate and lower in lipids and proteins, in contrast to newly introduced formulations. In order to evaluate current liver injury incidence and feeding adequacy, this study may have to be repeated.

## 5. Conclusions

Our findings suggest that liver test abnormalities are multifactorial in ICU patients treated with 3 or more consecutive days of PN. The presence of elevated liver test parameters at the start of PN treatment was revealed to be the most important factor, while sepsis and AHF were strong contributors to further liver test disturbances. PN and hepatotoxic drugs had a minor impact. This study demonstrates that close follow-up of aminotransferases is indicated in patients receiving PN with the simultaneous presence of AHF and/or sepsis and that patients can generally be safely treated with the preselected hepatotoxic drugs.

To be in line with ESPEN recommendations, carbohydrate intake should be reduced and lipid and protein intake should be increased in our patient population. In recent years, the ready-to-use multi-chamber PN bag composition has already changed to less carbohydrates and more lipids and proteins, which is in line with these findings. More research in vivo and in vitro is necessary to assess the influence of the individual PN components on liver parameters and to evaluate new PN formulations.

## Figures and Tables

**Table 1:** Baseline characteristics upon admission.

	No liver Disturbances (N = 162)	Liver Disturbances (N = 62)	Total (N = 224)	*p*-Value
**Baseline characteristics**				
Sex female—no. (%)	62 (38.3)	23 (37.1)	85 (37.9)	0.871
Age (y)	65 ± 16	60 ± 17	63 ± 17	0.040
Height (cm)	169 ± 10	172 ± 10	171 ± 10	0.196
Weight (kg)	76 ± 20	78 ± 15	77 ± 19	0.221
BMI	26 ± 6	26 ± 5	26 ± 6	0.700
Body Mass Index—no. (%)				0.224
<18.5	12 (7.4)	1 (1.6)	13 (5.8)	
18.5–25	61 (37.7)	27 (43.5)	88 (39.3)	
>25	89 (54.9)	34 (54.8)	123 (54.9)	
ABW (kg)	68 ± 10	71 ± 9	69 ± 10	0.078
LOS ICU (days)	16 [9–26]	18 [9–33]	19 [10–31]	0.243
APACHE II score	21 ± 11	24 ± 11	22 ± 11	0.048
28 days mortality—no. (%)	43 (26.5)	23 (37.1)	66 (29.5)	0.121
**Nutritional characteristics**				
PN therapy duration at ICU (days)	6 [4–12]	6 [4–11]	8 [5–14]	0.540
Tube feeding—no. (%)	93 (57.4)	41 (66.1)	134 (59.8)	0.234
TF therapy duration at ICU (days)	12 [6–23]	7 [4–28]	12 [6–23]	0.219
REE (Kcal)	1772 [1349–2225]	1877 [1689–2180]	1834 [1492–2139]	0.533
Calorie requirement (Kcal)	1708 ± 261	1764 ± 232	1732 ± 251	0.078
Amino acid requirement (g)	89 ± 14	92 ± 12	90 ± 13	0.078
Carbohydrate requirement (g)	137 ± 21	141 ±19	139 ± 20	0.078
Lipid requirement (g)	102 ± 16	106 ± 14	104 ± 15	0.078
IC performed—no. (%)	20 (12.3)	6 (9.7)	26 (11.6)	0.577
**PN intake**				
Volume of PN administered daily (mL)	1450 ± 388	1572 ± 439	1482 ± 406	<0.001
PN Calories administered daily (kcal)	1603 ± 548	1767 ± 499	1642 ± 472	<0.001
PN AA administered daily (g)	64 ± 22	71 ± 21	66 ± 18	<0.001
PN CH administered daily (g)	197 ± 69	217 ± 60	201 ± 62	<0.001
PN lipids administered daily (g)	58 ± 22	62 ± 17	59 ± 16	<0.001
**Co-morbidities**				
Sepsis/septic shock—no. (%)	35 (21.6)	22 (35.5)	57 (25.4)	0.033
Acute Heart Failure—no. (%)	19 (11.7)	18 (29)	37 (16.5)	0.002
**Hepatotoxic drugs**				
Paracetamol prescribed—no. (%)	151 (93.2)	59 (95.2)	210 (93.8)	0.589
Paracetamol therapy (days)	22 [14–36]	19 [9–35]	21 [12–36]	0.250
AC prescribed—no. (%)	114 (70.4)	40 (64.5)	154 (68.8)	0.398
AC therapy (days)	5 ± 4	4 ± 3	5 ± 4	0.157
Flucloxacillin prescribed—no. (%)	15 (9.3)	2 (3.2)	17 (7.6)	0.127
Flucloxacillin therapy (days)	10 ± 12	11 ± 8	9 ± 10	0.618
Valproic acid prescribed—no. (%)	11 (6.8)	2 (3.2)	13 (5.8)	0.307
Valproic acid therapy (days)	4 [4–24]	7 [4–7]	7 [4–20]	0.923
SMT/TMP prescribed—no. (%)	20 (16.1)	10 (16.1)	30 (13.4)	0.457
SMT/TMP therapy (days)	6 [2–9]	13 [4–22]	7 [2–13]	0.143

APACHE II score: minimum 0 and maximum 71; increasing score is associated with an increasing risk of hospital death, ABW: calculated when 18.5 > BMI > 25. Abbreviations: TF tube feeding; LOS length of stay; APACHE: Acute Physiology and Chronic Health Evaluation; BMI: body mass index; ABW: adjusted body weight; ICU: intensive care unit; PN: parenteral nutrition; IC: indirect calorimetry; REE: resting energy expenditure; AC: amoxicillin/clavulanic acid; SMT/TMP: sulfamethoxazole/trimethoprim; Kcal: kilocalories; AA: amino acids; CH: carbohydrates; Lip: lipids. Results expressed as no. (%), mean ± SD or median [IQR], *p* < 0.05 were considered significant.

## Data Availability

The datasets used and analyzed during this study are available from the corresponding author on reasonable request.

## Supplementary Materials

The following supporting information can be downloaded at: https://www.mdpi.com/article/10.3390/nu15112612/s1, Table S1: PN bag composition.

## Appendix A

Appendix A includes a table with the multivariable association between liver parameters and possible confounders based on their biologically plausible association.

**Table A1:** Multivariable associations between liver parameters (intercept) and possible confounders based on their biologically plausible association such as hepatotoxic drugs, co-morbidities, and PN in critically ill patients follow between 3 and 10 days. (EXP(estimate) − 1) × 100 in order to calculate increase or decrease (%).

	Estimate (SE)	Increase/Decrease (%)	**t Value**	***p* Value**
**AST (U/L) (3.81 ± 0.24 U/L)**				
Duration on ICU (per day)	−0.041 (0.009)	−4.0	−4.377	<0.001
Acute heart failure vs. no acute heart failure	0.561 (0.128)	75.2	4.377	<0.001
Cumulative dose of paracetamol (per g)	0.018 (0.004)	1.8	4.131	<0.001
Cumulative dose of amoxicillin/clavulanic acid (per g)	0.007 (0.005)	0.7	1.580	0.114
Cumulative dose of SMT/TMP (per g)	0.023 (0.010)	2.3	2.299	0.022
Liver dist at start PN vs. no liver dist at start PN	1.030 (0.106)	180.1	9.739	<0.001
Volume of PN (per L)	0.132 (0.059)	14.1	2.252	0.025
BMI (per point)	−0.013 (0.009)	−1.3	−1.574	0.117
**ALT (U/L) (4.29 ± 0.24 U/L)**				
Duration on ICU (per day)	−0.020 (0.008)	−2.0	−2.590	0.009
Age (per year)	−0.005 (0.003)	−0.5	−1.660	0.097
Cumulative dose of paracetamol (per g)	0.013 (0.004)	1.3	3.700	<0.001
Cumulative dose of amoxicillin/clavulanic acid (per g)	0.022 (0.004)	2.2	5.530	<0.001
Volume of PN (per L)	0.091 (0.050)	9.5	1.860	0.063
Acute heart failure vs. no acute heart failure	0.258 (0126)	29.4	2.040	0.043
Liver dist at start PN vs. no liver dist at start PN	1.090 (0.106)	197.4	10.300	<0.001
**AP (U/L) (4.06 ± 0.08 U/L)**				
Acute heart failure vs. no acute heart failure	−0.151 (0.093)	−14.0	−1.620	0.107
Duration on ICU (per day)	0.056 (0.005)	5.8	11.100	<0.001
Liver dist at start PN vs. no liver dist at start PN	0.371 (0.078)	44.9	4.760	<0.001
Cumulative dose of flucloxacillin (per g)	0.017 (0.004)	1.7	4.200	<0.001
Cumulative dose of amoxicillin/clavulanic acid (per g)	0.005 (0.003)	0.5	1.940	0.053
Cumulative dose of paracetamol (per g)	0.017 (0.002)	1.7	6.930	<0.001
Sepsis/septic shock vs. no sepsis/septic shock	0.281 (0.079)	32.4	3.580	<0.001
Male	0.125 (0.071)	13.3	1.770	0.077
Volume of PN (per L)	−0.024 (0.033)	−2.4	−0.742	0.458
**GGT (U/L) (3.41 ± 0.10 U/L)**				
Sepsis/septic shock vs. no sepsis/septic shock	0.304 (0.129)	35.5	2.360	0.019
Male	0.231 (0.115)	26.0	2.000	0.046
Liver dist at start PN vs. no liver dist at start PN	0.519 (0.125)	68.0	4.150	<0.001
Cumulative dose of SMT/TMP (per g)	−0.017 (0.009)	−1.7	−1.810	0.071
Cumulative dose of valproic acid (per g)	−0.057 (0.041)	−5.5	−1.390	0.164
Cumulative dose of flucloxacillin (per g)	0.013 (0.006)	1.3	2.230	0.026
Cumulative dose of amoxicillin/clavulanic acid (per g)	0.022 (0.004)	2.2	4.930	<0.001
Cumulative dose of paracetamol (per g)	0.026 (0.004)	2.6	6.760	<0.001
Duration on ICU (per day)	0.130 (0.008)	13.9	16.200	<0.001
**INR (−1.01 ± 0.10)**				
Duration on ICU (per day)	−0.030 (0.004)	−3.0	−8.250	<0.001
Liver dist at start PN vs. no liver dist at start PN	0.112 (0.047)	11.9	2.380	0.018
Sepsis/septic shock vs. no sepsis/septic shock	0.217 (0.048)	24.2	4.520	<0.001
BMI (per point)	0.010 (0.004)	1.0	2.620	0.010
Cumulative dose of SMT/TMP (per g)	−0.006 (0.004)	−0.6	−1.540	0.123
Cumulative dose of amoxicillin/clavulanic acid (per g)	0.004 (0.002)	0.4	2.260	0.024
Cumulative dose of paracetamol (per g)	0.013 (0.002)	1.3	7.440	<0.001
**TB (mg/dL) (0.52 ± 0.05 mg/dL)**				
Duration on ICU (per day)	0.019 (0.003)	1.9	5.860	<0.001
Cumulative dose of valproic acid (per g)	−0.031 (0.020)	−3.1	−1.540	0.125
Cumulative dose of flucloxacillin (per g)	0.010 (0.003)	1.0	3.840	<0.001
Cumulative dose of amoxicillin/clavulanic acid (per g)	0.003 (0.002)	0.3	1.680	0.094
Cumulative dose of paracetamol (per g)	−0.009 (0.002)	−0.9	−5.540	<0.001
Liver dist at start PN vs. no liver dist at start PN	0.357 (0.061)	42.9	5.770	<0.001
Sepsis/septic shock vs. no sepsis/septic shock	0.356 (0.063)	42.8	5.600	<0.001
Volume of PN (per L)	0.001 (0.021)	0.1	0.049	0.961

Results expressed as estimate, standard error (SE), increase/decrease (%), t-value, and *p*-value. Abbreviations: AST: aspartate aminotransferase; ALT: alanine aminotransferase; AP: alkalic phosphatase; GGT: gamma-glutamyl transferase; INR: international normalized ratio; TB: total bilirubin; SMT/TMP: sulfamethoxazole/trimethoprim; ICU: intensive care unit; BMI: body mass index.

## Appendix B

Appendix B includes a table about the multivariable association between AST and possible confounders.

Table A2 shows four different situations in which the influence of the different confounders is clearly evident. AST has a mean value of 3.81 U/L (estimate) when all confounders are negative.

Situation 1: A patient with AHF and liver disturbances at the start of PN therapy (cause could be AHF or other) treated with 4 g of paracetamol and a BMI of 25 at ICU day 3 but who did not receive amoxicillin/clavulanic acid, SMT/TMP, or PN.

Situation 2: Same as Situation 1, but treated with 2L PN.

Situation 3: Same patient as Situation 2, but without AHF.

Situation 4: Same patient as Situation 3, but without AHF and no liver test disturbances at the start of PN therapy.

**Table A2:** Multivariable association between AST and possible confounders.

Situation 1		Situation 2		Situation 3		Situation 4	
Parameter	=e(estimate)	Parameter	=e(estimate)	Parameter	=e(estimate)	Parameter	=e(estimate)
AST	=e(3.81)	AST	=e(3.81)	AST	=e(3.81)	AST	=e(3.81)
AHF (yes)	=e(0.561)	AHF (yes)	=e(0.561)	AHF (no)	N/A	AHF (no)	N/A
LD (yes)	=e(1.03)	LD (yes)	=e(1.03)	LD (yes)	=e(1.03)	LD (no)	N/A
PCT (g)	=e(4 × 0.018)	PCT (g)	=e(4 × 0.018)	PCT (g)	=e(4 × 0.018)	PCT (g)	=e(4 × 0.018)
BMI	=e(−0.013 × 25)	BMI	=e(−0.013 × 25)	BMI	=e(−0.013 × 25)	BMI	=e(−0.013 × 25)
Day ICU	=e(−0.041 × 3)	Day ICU	=e(−0.041 × 3)	Day ICU	=e(−0.041 × 3)	Day ICU	=e(−0.041 × 3)
PN (L)	N/A	PN (L)	=e(2 × 0.132)	PN (L)	=e(2 × 0.132)	PN (L)	=e(2 × 0.132)
AST	152.2 U/L	AST	198.1 U/L	AST	113.1 U/L	AST	40.4 U/L

Abbreviations: AST: aspartate aminotransferase; AHF: acute heart failure; LD: liver disturbances at start of PN therapy; PCT: paracetamol; BMI: body mass index; ICU: intensive care unit; PN: parenteral nutrition.
