# Supplementary material for: Parenteral Nutrition, Sepsis, Acute Heart Failure and Hepatotoxic Drugs Are Related to Liver Test Disturbances in Critically Ill Patients
Source: Nutrients. 2023 Jun 2;15(11):2612. doi: 10.3390/nu15112612 (PMC10255719; doi:10.3390/nu15112612)
Supplement: Supplementary file 1 [file nutrients-15-02612-s001.zip › nutrients-2393675-supplementary.pdf]

Supplementary Materials

Table S1 includes a table regarding the composition of PN bags administered during this study.

Table S1: PN bag composition

**Composition**	**N4E**	**N5E**	**N7E**	**N7E**	**N9E**	**SMOFkabiven**	**Schema A (fluid restriction)**	**Schema C(less glucose)**
**Volume (mL)**	1500	2000	1500	2000	1000	2463	1265	2073
**Aminoacids (g)**	37.9	65.8	66.4	88.6	56.9	123	56.9	114
**Glucose (g)**	113	230	210	280	110	313	275	149
**Lipids (g)**	45	80	60	80	40	94	51	104
**Calories (kcal)**	1050	1980	1710	2270	1070	2700	1505	1596

Abbreviations: N nitrogen, number grams of nitrogen/liter PN, SMOF soy bean oil/middle chain triglycerides/Olive oil/Fish oil
